# Elevated levels of aquaporin-4-containing extracellular vesicles in cerebrospinal fluid of patients with bipolar disorder

**DOI:** 10.48101/ujms.v130.12006

**Published:** 2025-03-21

**Authors:** Lennart Wetterberg, Fariborz Mobarrez, Rolf Nybom, Håkan Wallén, Aurimantas Pelanis, Dietrich von Rosen, Mikael Landén

**Affiliations:** a Department of Clinical Neuroscience, Karolinska Institutet, Stockholm, Sweden; b Department of Medical Sciences, Uppsala University, Akademiska Hospital, Uppsala, Sweden; c Department of Clinical Sciences, Danderyd Hospital, Division of Cardiovascular Medicine, Karolinska Institutet, Stockholm, Sweden; d Department of Anesthesiology, Sahlgrenska University Hospital, Gothenburg, Sweden; e Department of Mathematics, Linköping University, Linköping, Sweden; f Institute of Neuroscience and Physiology, The Sahlgrenska Academy at Gothenburg, University, Gothenburg, Sweden; g Department of Medical Epidemiology and Biostatistics, Karolinska Institutet, Stockholm, Sweden

**Keywords:** Aquaporin-4, extracellular vesicles, cerebrospinal fluid, flow cytometry

## Abstract

**Objectives:**

To examine a hypothetical dysfunction of the brain water channels in bipolar disorder by analyzing aquaporin-4 (AQP4) exposing extracellular vesicles (EVs) in cerebrospinal fluid (CSF) from individuals with bipolar disorder types 1 and 2, and healthy controls.

**Methods:**

We analyzed exposure of AQP4 EVs to three different epitopes – the N- and C-terminals, and the epitope containing amino acids 273–291 – in CSF by flow cytometry in 24 individuals with bipolar disorder (type 1, *n* = 20; type 2, *n* = 4) and in 14 healthy controls.

**Results:**

We observed significantly higher levels of EVs expressing AQP4 in the CSF from individuals with bipolar disorder compared with healthy controls. Specifically, the mean ± SD concentration of AQP4 + EVs per μl CSF for the N-terminal epitope was 346 ± 22 in patients with bipolar disorder type 1, 386 ± 78 in those with bipolar disorder type 2, compared with 39 ± 6.9 in the healthy control group (*P* < 0.0001). For AQP4+ EVs targeting the C-terminal epitope, the corresponding values were 350 ± 22 for bipolar disorder type 1, 374 ± 46 for bipolar disorder type 2, and 36 ± 6.3 for healthy controls. Similarly, EVs expressing AQP4+ epitopes containing amino acids 273–291 showed concentrations of 344 ± 17 in bipolar disorder type 1, 398 ± 63 in bipolar disorder type 2, and 38 ± 6.4 in the control group (*P* < 0.0001).

**Conclusion:**

Our findings revealed significantly more EVs expressing the three AQP4 epitopes in patients with bipolar disorder compared with healthy controls. This suggests a dysregulated expression of AQP4, implicating a potential disruption in brain water homeostasis as a contributing pathogenic mechanism in bipolar disorder.

## Introduction

Bipolar disorder is a complex psychiatric condition characterized by extreme mood fluctuations, ranging from manic highs to depressive lows, and is associated with significant cognitive, emotional, and functional impairments. Despite decades of research, the precise biological mechanisms underpinning bipolar disorder remain poorly understood.

Increasing scientific attention has focused on the brain’s fluid dynamics and the roles played by the glymphatic system, extracellular vesicles (EVs), and the water channel protein aquaporin-4 (AQP4) in maintaining neural health and homeostasis (1, 2). These systems are critically involved in the regulation of brain waste clearance, cellular communication, and neuroinflammation – key processes that may be disrupted in bipolar disorder.

The nanoscale EVs, ranging from 0.1 to 1 μm in diameter, are released from their mother cells in response to various forms of cellular stress, such as inflammation, infection, toxin exposure, or apoptosis. A potential disturbance of AQP4 water channels could be identified through the analysis of EVs in the cerebrospinal fluid (CSF).

**Figure 1:**
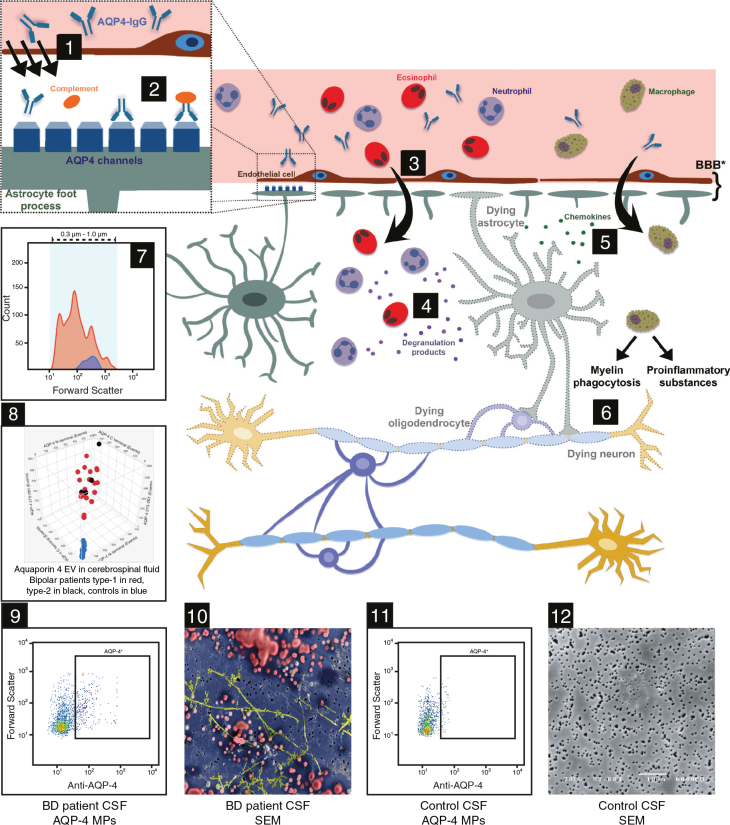
A montage of hypothetical pathogenesis of aquaporin-4 extracellular vesicles (EVs) (or microvesicles) in bipolar disorders (number 1–6) and clinical findings in cerebrospinal fluid (CSF) in the present study of 24 patients with bipolar disorder and 14 healthy controls (number 7–12). Aquaporin 4 (AQP-4)-IgG accesses the CNS at areas of increased blood-brain barrier (BBB) permeability or injury or across endothelial cells by transcytosis. The antibody binds selectively to AQP-4 antigen on astrocyte foot processes. The blood-brain barrier is formed by various components, some of which are illustrated: endothelial cells and astrocyte foot processes. 2. The antigen-antibody binding leads to complement activation and downregulation of the AQP-4 water channel. 3. Activated complement increases blood-brain barrier permeability and leads to leukocyte infiltration, particularly neutrophils and eosinophils. 4. Degranulation products and extracellular vesicles (EVs) (MPs) in CSF indicate astrocyte damage. 5. Chemokines and extracellular vesicles (EVs) are released from leukocytes and damaged astrocytes and attract macrophages. 6. Macrophages produce proinflammatory substances and phagocytose matter resulting in damage of oligodendrocytes and neurons. 7. Distribution of extracellular vesicles (EVs) in CSF, regardless of expression and phenotype, in 24 bipolar patients (red) and in 14 healthy controls (blue). Forward scatter on the X-axis indicates the size of the particles, and count (number of events as detected by the flow cytometer) on the Y-axis. 8. A 3D diagram of three epitopes of aquaporin 4^+^ EV in cerebrospinal fluid in 24 bipolar patients type 1 in red or bipolar patients type 2 in black and in 14 healthy controls indicated in blue dots. For specificities of the three antibodies of aquaporin 4 used in this study, see Table 2. As noticed, there is a clear delineation with no overlaps between patients and controls in numbers of all three AQP-4^+^ EVs. 9. Representative flow cytometry plot demonstrating AQP-4^+^ extracellular vesicles (EVs) in the CSF in a patient with bipolar disorder. Forward Scatter of EVs on the Y-axis (i.e. size of particles) and Anti-AQP-4 FITC binding on the X-axis. 10. Photo of scanning electron microscopy (SEM) of CSF on the polycarbonate filter (pore diameter 0.6 μm) displays spherical EVs and other unidentified structures from the degranulation processes. 11. Representative flow cytometry plot demonstrating AQP-4+ EVs in CSF in a control subject. Forward Scatter of EVs on the Y-axis (i.e. size of particles) and Anti-AQP-4 FITC binding on the X-axis. 12. Photo of SEM of CSF from a healthy control did not reveal any EVs on the polycarbonate filter. The many small black structures in the filter indicate open pores with a diameter of 0.6 μm. The cartoons 1–6 are adapted with permission from Dutra BG, da Rocha AJ, Nunes RH, et al. *RadioGraphics*. 2018;38(1):169–193.

In 2010, a study using scanning microscopic technology identified previously undescribed microparticles in CSF from patients with bipolar disorder (see Figure 1 illustration nr 10) (3). At that time, we hypothesized that ‘the number of microscopic structures in the CSF, from none to many, might correlate with the degree of the purported underlying disease processes in the central nervous system in patients with bipolar disorder’.

In 2013, Nagelhus and Ottersen reported that AQP4 was the most common water channel in the astrocytes (4). In 2019, Bejerot and colleagues reported abnormally high levels of AQP4+ EVs in CSF sampled from a 22-year-old female patient with several symptoms of an affective disorder, 2 years before her full clinical presentation of neuromyelitis optica. This was later confirmed by elevated titers of AQP4 antibodies in her serum (5). This observation indicates that an increase in AQP4+ EV in the central nervous system, detectable in the adjoining CSF, could serve as an early indicator of pathophysiological changes involving the AQP4 mechanisms.

**Table 1:** Characteristics of bipolar patients and controls in the study of AQP4 extracellular vesicles in cerebrospinal fluid as *N* (%) or mean ± SD and (range).

Contents	Bipolar 1 *n* = 20	Bipolar 2 *n* = 4	Controls *n* = 14	*P* value bipolar disorder 1 & 2 versus controls
Sex, female	11 (55)	4 (100)	8 (57)	NS
Age years	48.1 ± 18 (22–79)	56.3 ± 18 (40–74)	45.4 ± 15.3 (23–64)	NS
Age of onset in years	24.3 (12–54)	14.8 (12–20)	-	
GAF symptom	67.8 ± 12.2 (35–90)	65 ± 10.8 (55–80)	88.7 ± 3.2 (85–91)	*P* < 0.025
GAF function	68.4 ± 11.6 (45–90)	66 ± 10.3 (55–80)	88.7 ± 3.2 (85–91)	*P* < 0.05
*Laboratory data*				
CSF/serum albumin ratio	5.7 ± 2.6 (3.1–12.5)	5.3 ± 2.8 (2.7–9.2)	4.5 ± 1.6 (3.1–8.5)	NS
Erythrocytes in CSF, nr of cells	<5	<5	<5	
*Medication*				
Lithium	18 (90)	0	0	
Valproate	2	1	0	
Lamotrigine	2	1	0	
Carbamazepine	0	1	0	
Antidepressants	5	3	0	
Somatic medication	7	3	4	

SD: standard deviation; GAF: Global Assessment of Functioning; CSF: cerebrospinal fluid.

**Table 2:** Aquaporin 4 (AQP4) antibodies used in this study of extracellular vesicles in cerebrospinal fluid of bipolar patients.

Antibodies	Epitope amino acids in the antibodies	Article number	Purchased from
AQP4 N-terminal	N-terminal	ABIN1838149	Antibodies-online
AQP4 C-terminal	C-Terminal	ABIN11637	LSBio Inc. Seattle, WA, USA
AQP4 epitope aa 273–291	TKGS^276^YMEVEDNRS^285^QVETDD	LS-C229824-100)	LSBio Inc. Seattle, WA, USA

In 2020, Gur et al. conducted the first investigation of the prevalence of serum AQP4-IgG in patients with bipolar disorder (6). But contrary to their hypothesis, no AQP4 autoantibodies were detected in the serum from any of their 25 bipolar disorder patients, neither at baseline nor at a follow-up assessment. Despite this, the authors concluded that ‘AQP4 may still play a role in the pathogenesis of mood disorders through various mechanisms of action such as altered AQP4 expression in the brain’. They suggested that CSF should be examined to further investigate AQP4-related pathology in patients with bipolar disorders.

The aim of this study was to investigate the potential role of AQP4 dysfunction in the pathophysiology of bipolar disorder. To this end, we analyzed the number of AQP4+ EVs in the CSF of a well-characterized cohort of bipolar disorder patients and in the CSF from healthy controls.

## Patients and methods

### Ethics

All participating patients and control subjects consented orally and in writing to participate in the study. The project was approved by the Stockholm Regional Ethical Review Board and conducted in accordance with the Helsinki Protocol (case no. 2005/554-31/3 and 2009/1221-32).

### Patients

The study setting was the bipolar outpatient unit at the Northern Stockholm psychiatric clinic, previously reported as the St. Göran study (7). In summary, outpatients referred for treatment and continuing patients were invited to participate in the present project provided they were at least 18 years old and met the diagnostic criteria for bipolar disorder according to the Diagnostic and Statistical Manual of Mental Disorders, Fourth Edition (DSM-IV) (8). In addition, the structured psychiatric interview Mini International Neuropsychiatry Interview (M.I.N.I.) was used to screen for and diagnose comorbid psychiatric diagnoses (9). Details of the study setting have been previously reported (3).

A total of 24 patients (15 females) with bipolar disorder, aged 22–79 years, were included in this study. Among them, 20 patients were diagnosed with bipolar disorder type 1 (11 females) and 4 with type 2 (all females). Patients with bipolar disorder types 1 and 2 were analyzed as separate diagnostic groups. All patients were in stable mood state and on stable treatment regimen at the time of lumbar puncture for CSF. Of the 24 patients, CSF samples from 22 patients were collected as part of a 7-year follow-up program, while 2 samples were collected at baseline examinations.

### Healthy controls

The control group was randomly selected by Statistics Sweden (SCB) and contacted via mail. Individuals who volunteered to participate were subsequently contacted by a research nurse for a preliminary telephone screening to exclude those with a history of mental illness, neurological problems, or substance abuse. Eligible participants were scheduled for a 1-day comprehensive assessment, during which they underwent a psychiatric evaluation by experienced clinicians using the M.I.N.I. Neuropsychiatric interview to rule out psychiatric disorders (9). Additional exclusion criteria were neurological conditions other than mild migraines, untreated endocrine disorders, pregnancy, dementia, and a family history of schizophrenia or bipolar disorder in first-degree relatives. For this study, CSF samples from 14 controls (8 females) collected as part of the St. Göran project, Sweden, were analyzed for AQP4 EVs.

### Collection of CSF

Lumbar punctures were performed in a sitting position between 09:00 and 10.30 AM, using fine disposable needles (Becton Dickinson 22 GA 3.00 IN 0.7 × 75 mm) and standardized procedures for all participants. The skin in the lumbar region was thoroughly washed with sterile cotton swabs and chlorhexidine solution (5 mg/mL, Fresenius Kabi) before puncture. The needle was inserted into the L3-L4, or L4-L5 vertebral interspace.

For the present analyses, after the initial 14 mL CSF had been collected for biobank storage pending other analyses, the last 6–12 drops were collected (approximately 0.3 mL) in sterile test tubes from each of the 24 patients the 14 controls. None of the samples were centrifuged, ensuring that no brain-derived components in CSF were pelleted or lost due to uneven distribution within the sample. The collected samples were stored at -80°C within less than 1 h of the lumbar punctures, preserving them for subsequent analyses of EVs.

### Analysis of blood–CSF barrier function

The blood-CSF barrier function was assessed by calculating the ratio between albumin concentration in CSF (mg/L) to serum (g/L). Albumin concentrations in both CSF and serum were measured by immunonephelometry on a Beckman Image Immunochemistry system (Beckman Instruments, Beckman Coulter, Brea, CA, USA) at the Clinical Neurochemistry Laboratory in Mölndal, Sweden. This method is accredited by the Swedish Board for Accreditation and Conformity Assessment (SWEDAC).

### Flow cytometric analyses of AQP4 EVs in CSF

Non-centrifuged CSF samples were stored at -80°C for approximately 2 years pending analyses. For preparation, frozen CSF samples were thawed in a 37°C water bath for approximately 5 min (10). An initial centrifugation step was conducted at 2000 × g for 20 min at room temperature to remove large debris. The resulting supernatant was carefully transferred to new tubes.

A 20 μL aliquot of the supernatant was incubated in the dark for 20 min with 5 μL of anti-AQP4 antibodies (Antibodies Online), targeting either the C-terminal epitope (ABIN11637), the N-terminal epitope (ABIN1838149), or the AQP4 epitope spanning amino acids 273–291 (see Table 2 for details). All antibodies were FITC-labeled (FITC Conjugation Kit [Fast] – Lightning, Abcam, Cambridge, UK).

For flow cytometric analysis, the samples were diluted with 120 μL of CytoFLEX Sheath Fluid (Beckman Coulter, Brea, CA, USA) and analyzed using a CytoFLEX flow cytometer (Beckman Coulter). Gating parameters were established with Nano fluorescent Yellow Particles with sizes of 0.13 μm, 0.22 μm, 0.45 μm, 0.88 μm, and 1.35 μm (Spherotech, Lake Forest, IL, USA), covering a detection range from approximately 0.2 μm to 1.0 μm. The lower EV gate was set at 0.2 μm to minimize background noise.

The gating strategy, based on side scatter and forward scatter, was further validated using both labeled and unlabeled EVs, accounting for differences in refractive indices between beads and EVs. Instrument calibration included unstained EVs, isotype controls, and single fluorochrome-stained EVs. The threshold was set to violet side scatter, and astrocyte-derived EVs were identified as those expressing AQP4.

Results are reported as the number of EVs per microliter of CSF, based on the 20 μL supernatant. The intra- and inter-assay coefficients of variation for this flow cytometric analysis were maintained at less than 10%.

### Statistical analysis

ANOVA, Kruskal-Wallis, and Wilcoxon´s tests were used to identify differences between the healthy controls and the type 1 and 2 bipolar disorder groups. In all statistical analyses, significance was set at *P*-values < 0.05. The SAS/STAT^®^ software was used for all calculations. Values are expressed as mean ± SD.

## Results

### Demographics

The study included 24 patients with bipolar disorder with a mean age of 48 years (range 22–79) and a mean age of onset of 24 years (range 12–54). Twenty patients were diagnosed with type 1 and 4 with type 2 bipolar disorder. The healthy control group consisted of 14 persons with a mean age of 45 years (range 23–64), none of whom had been treated for depression, mania/hypomania, schizophrenia, or other psychoses.

The characteristics of the patients and controls are presented in Table 1, which shows that patients had a greater global symptom burden and lower global functioning than controls. With respect to medication, 90% of the bipolar disorder type 1 group were prescribed lithium, while none of the bipolar disorder type 2 patients were prescribed lithium.

The CSF/serum albumin ratio was numerically somewhat higher in the bipolar disorder group, with mean ± SD values of 5.7 ± 2.6 for type 1 and 5.3 ± 2.8 for type 2, compared to 4.5 ± 1.6 in healthy controls. However, this difference did not reach statistical significance (*P* = 0.16).

### The AQP4 EVs in the CSF

The AQP4^+^ EVs were identified by size (forward scatter and side scatter) and expression of AQP4+. The number of EVs expressing AQP4, detected using antigens targeting the three epitopes (see Table 2), was significantly higher in the CSF of bipolar disorder patients compared with the healthy controls.

Specifically, the mean ± SD concentration of AQP4^+^ EVs/μl CSF for the N-terminal epitope was 346 ± 22 in bipolar disorder type 1 patients and 386 ± 78 in bipolar disorder type 2 patients, compared with 39 ± 6.9 in the healthy control group (*P* < 0.0001). For the C-terminal epitope, the corresponding values were 350 ± 22 for bipolar disorder type 1, 374 ± 46 for bipolar disorder type 2, and 36 ± 6.3 in the control group (*P* < 0.0001). Similarly, for the epitope containing amino acids 273–291, the concentrations were 344 ± 17 for bipolar disorder type 1, 398 ± 63 for bipolar disorder type 2, and 38 ± 6.4 in the control group (*P* < 0.0001). The range of values is detailed in Table 3, and a 3D graphic display of all three AQP4^+^ EVs epitopes is provided in Figure 1, illustration 8.

**Table 3:** Events of extracellular vesicles (EVs) exposing aquaporin 4 (AQP4) in cerebrospinal fluid expressed as mean, standard error and (range).

Antibodies of AQP*4*-FITC	Bipolar 1 *n* = 20, 11 F†	Bipolar 2 *n* = 4, 4 F	Controls *n* = 14, 8 F	*P* value bipolar disorder 1 and bipolar disorder 2 versus controls Both ANOVA and Kruskal-Wallis test
AQP4+ EVs N-terminal	346 ± 22 (162–514)	386 ± 78 (272–606)	39 ± 6.9 (8–78)	*P* < 0.0001
AQP4+ EVs C-terminal	350 ± 22 (175–560)	374 ± 46 (294–507)	36 ± 6.3 (6–77)	*P* < 0.0001
AQP 4+ EVs Amino acids 273-291	344 ± 17 (199–479)	398 ± 63 (306–583)	38 ± 6.4 (7–84)	*P* < 0.0001

† F: females. For individual results of AQP4 EVs, see Figure 1, image nr 8, 3D Scatter plot values.

The group difference of AQP4^+^ EVs remained across all age groups and was independent of the number of disease episodes. Notably, this pattern was observed even in the two youngest patients, whose bipolar symptoms emerged at ages 22 and 28 years. Intriguingly, there was no overlap in AQP4^+^ EVs levels between the patient and control groups, as the maximal value recorded for any of the 14 controls was lower than the minimal value observed for any of the 24 patients.

## Discussion

Several previous studies have explored biological markers in CSF from the overarching case-control cohort from which we recruited patients (11–13). In the present study, the aim was to elucidate whether case-control differences in AQP4^+^ EVs levels in CSF may provide novel insight into the pathophysiology of bipolar disorder.

Our findings demonstrated significant elevation of AQP4+ EVs in the CSF of individuals with bipolar disorder type 1 and 2 as compared to healthy controls. This suggests that AQP4+ EVs may hold potential as a diagnostic or prognostic risk marker for bipolar disorder. However, the underlying mechanism driving this elevation remains to be investigated. Possible explanations include genetic variation in the AQP-4 gene, or a disruption of the blood-brain barrier, both of which warrant further investigation.

### Aquaporin-4

The properties of water channels, particularly AQP4, are of significant interest in psychiatric disorders due to the abundance of AQP4 tetramers at the blood-brain interface. The term ‘interface’ is used here to reflect the positioning of the astrocytic end-feet, which lie outside of the traditionally defined blood-brain barrier (see Figure 1, illustrations 1 and 2).

The significantly higher levels of mean of AQP4^+^ EVs in the bipolar disorder group compared to controls raises the question if this could be caused by variation in the regulation of the AQP-4 gene. A disrupted switching mechanism in astrocytes could contribute to the clinical presentation, including the sometimes rapid cycling between manic and depressive episodes observed in some patients with bipolar disorder. The ability of AQP4^+^ EVs to cross the blood-brain barrier, moving between the CNS and the peripheral circulation, has increased the interest in EV research within the psycho-neurological fields. It is even possible that AQP4^+^ EVs may not only be associated with bipolar disorder, but could also play a role in the pathophysiology of other neuropsychiatric diseases (14).

### Strengths of the study

The study benefits from highly standardized laboratory methods and well-characterized patient and control groups. All analyses were conducted in a laboratory using consistent assay lots and performed by a technician experienced in EV analysis in CSF.

### Limitations

A key limitation is the lack of investigation into bipolar disorder patients during unmedicated episodes of both manic and depressive stages. This gap leaves open questions about how mood states may influence the presence and behavior of AQP4+ EVs.

### Potential implications

Understanding the role and molecular mechanisms of AQP4+EVs could provide novel therapeutic targets, particularly for treatments aimed at restoring blood-brain barrier (BBB) integrity in bipolar disorder. Such approaches might include the use of stem cells, biomaterials, anti-inflammatory drugs, and the development of mood stabilizers that focus on BBB repair and preservation (15).

## Conclusion

**Figure 2:**
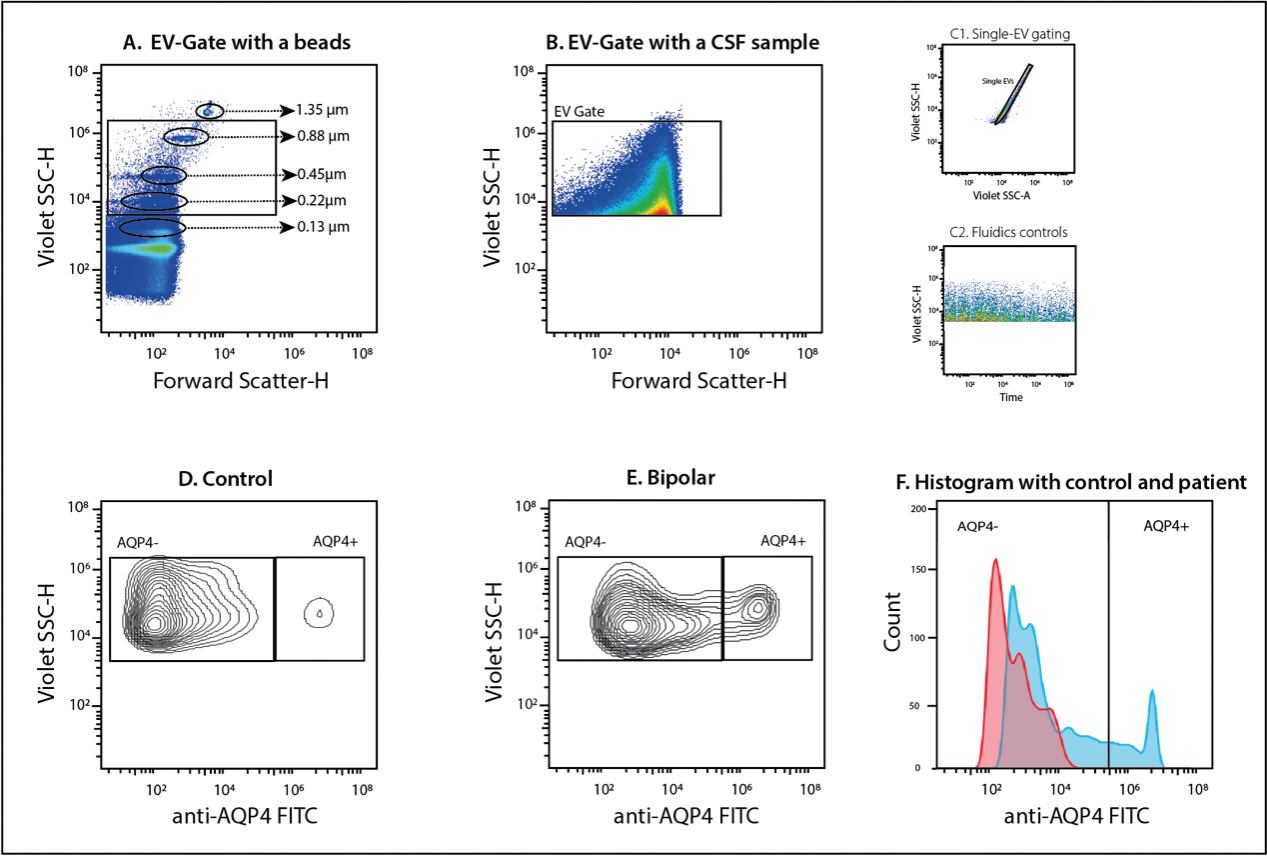
Representative plots of flow cytometric analysis of extracellular vesicles (EVs) in cerebrospinal fluid (CSF) stained with anti-AQP4 FITC targeting the N-terminal epitope. Nano Fluorescent Yellow Particles (0.13–1.35 μm) were used to define the EV gate based on size (Forward Scatter-H) and complexity (Violet SSC-H) (A). A CSF sample gated for EVs is shown, with events captured within the designated EV size range (B). To ensure accurate measurements, single-EV gating was applied to exclude doublets (C1), and fluidics controls were used to confirm sample acquisition stability (C2). A representative contour plot of a control CSF sample (D) demonstrates low levels of AQP4+ EVs, while a bipolar disorder patient sample (E) shows increased AQP4+ EVs. Finally, an overlay histogram (F) illustrates the separation between AQP4- and AQP4+ EV populations in a control sample (red) and a bipolar disorder patient (blue).

This study identified a significant increase in brain-derived EVs expressing AQP4 in the CSF of patients with bipolar disorder, compared to healthy controls. The observed dysfunction may be influenced by different isoforms of AQP4, which will be further explored (16). Representative flow cytometry plots illustrating the gating strategy, single-EV detection, and comparison between control and bipolar disorder samples are shown in Figure 2. Given the cyclical nature of bipolar disorder, including instances of spontaneous remission in untreated patients, further investigation into the role of AQP4 isomeric EVs in the disease process is warranted.
